# Detection of Heroin and Noscapine in Bile Specimen in a Body Packer

**Published:** 2019-10

**Authors:** Nahid NAJAFI, Mahmoud MONTAZERI

**Affiliations:** 1. Department of Pharmacodynamics and Toxicology, School of Pharmacy, Mashhad University of Medical Sciences, Mashhad, Iran; 2. Student Research Committee, Mashhad University of Medical Sciences, Mashhad, Iran; 3. Department of Toxicology and Pharmacology, School of Pharmacy, Shiraz University of Medical Sciences, Shiraz, Iran; 4. Department of Autopsy, Fars Province General Office, Legal Medicine Organization, Shiraz, Iran

## Dear Editor-in-Chief

"Body packers," also called "mules," are people who pack their gastrointestinal tract with bags of heroin, cocaine, amphetamines, and cannabis to smuggle the illegal drug from one country to another (1).

**Fig. 1:**
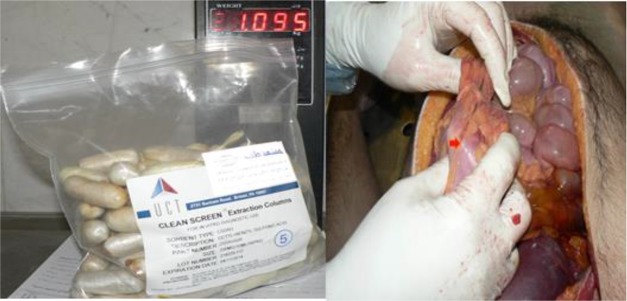
Autopsy of gastrointestinal & 97 packets recovered

In the present study, the deceased was a 21-yr-old Afghani male (height: 170 cm; weight: 65 kg) and Iranian nationals. He was found inappropriate at street via police officers about 12 h after last seen by the family, and according to his family, he addicted. He referred to the clinic, but he was very intoxication and also died the deceased was transferred to the Legal Medicine Organization of Shiraz for a medico-legal autopsy. The autopsy was performed six hours after the body found. Due to the identification performed autopsy for the deceased and viscera specimen (bile, liver, gastric contents) and urine sample sent to the toxicology laboratory, also performed amphetamine test addition of toxicology routine tests (alcohol, narcotic and drug analysis). There were no violent wounds found on the body or injection sites. An autopsy from the deceased revealed skin congestion and bleeding and congestion of the conjunctiva and pulmonary edema and congestion observed in both lungs. In the stomach, there were multiple condoms content of white powder (Fig. 1). No other remarkable change found in any other internal organ.

Autopsy performed within gastrointestinal, tube-shaped packages were found in the stomach. Overall, 97 complete packages and some empty plastic open and scattered that is not recoverable were found in the stomach and totally, the weight of them was 1.095 grand one of them for detection of content sent to the toxicology laboratory. Sample of the creamy powder obtained in the package of tube-shape from the autopsy analyzed via high-performance liquid chromatography (HPLC), and finally, heroin and noscapine detected. The cause of death in this victim was a heroin overdose due to rupturing one heroin package in the stomach.

In certain cases, an enormous amount of drug released when a packet, heroin well absorbed from the gastrointestinal tract. The autopsy findings, in this case, revealed cyanosis and pulmonary edema and congestion. Heroin and its metabolites were detected in the gastric contents. The estimated quantity of heroin that leaked into the stomach was 3.5 gm.

Heroin well absorbed from the gastrointestinal tract. Symptoms and signs of opiate toxicity include nausea, vomiting, constipation, depression of consciousness, respiratory depression, coma, and death (1–3). Tissue redistribution of heroin and its metabolites is very rapid (4, 5). This case of fatal heroin, due to ruptured body packets, was reported from the Department of Forensic Medicine. The powder was packed inside condoms without extra covering, contrary to professional packers. A significant level of heroin metabolites, noscapine was detected in the blood and urine. The cause of death was a heroin overdose.
